# A Comparison of the Predictive Value of the Glasgow Coma Scale and the Kampala Trauma Score for Mortality and Length of Hospital Stay in Head Injury Patients at a Tertiary Hospital in Uganda: A Diagnostic Prospective Study

**DOI:** 10.1155/2020/1362741

**Published:** 2020-10-13

**Authors:** Herbert Ariaka, Joel Kiryabwire, Ssenyonjo Hussein, Alfred Ogwal, Emmanuel Nkonge, Felix Oyania

**Affiliations:** ^1^Department of Surgery, Uganda Heart Institute, P.O 37392, Kampala, Uganda; ^2^Department of Surgery, College of Health Sciences, Makerere University, P.O. Box 7060, Kampala, Uganda; ^3^Department of Surgery, St Joseph's Hospital, Maracha, P.O. Box 59, Arua, Uganda; ^4^Department of Surgery, Kitovu Hospital, Masaka, P.O. Box 413, Masaka, Uganda; ^5^Department of Surgery, Mbarara Regional Referral Hospital, Mbarara, P.O. Box 1410, Mbarara, Uganda

## Abstract

**Introduction:**

The prevalence rates of head injury have been shown to be as high as 25% among trauma patients with severe head injury contributing to about 31% of all trauma deaths. Triage utilizes numerical cutoff points along the scores continuum to predict the greatest number of people who would have a poor outcome, “severe” patients, when scoring below the threshold and a good outcome “non severe” patients, when scoring above the cutoff or numerical threshold. This study aimed to compare the predictive value of the Glasgow Coma Scale and the Kampala Trauma Score for mortality and length of hospital stay at a tertiary hospital in Uganda.

**Methods:**

A diagnostic prospective study was conducted from January 12, 2018 to March 16, 2018. We recruited patients with head injury admitted to the accidents and emergency department who met the inclusion criteria for the study. Data on patient's demographic characteristics, mechanisms of injury, category of road use, and classification of injury according to the GCS and KTS at initial contact and at 24 hours were collected. The receiver operating characteristics (ROC) analysis and logistic regression analysis were used for comparison.

**Results:**

The GCS predicted mortality and length of hospital stay with the GCS at admission with AUC of 0.9048 and 0.7972, respectively (KTS at admission time, AUC 0.8178 and 0.7243). The GCS predicted mortality and length of hospital stay with the GCS at 24 hours with AUC of 0.9567 and 0.8203, respectively (KTS at 24 hours, AUC 0.8531 and 0.7276). At admission, the GCS at a cutoff of 11 had a sensitivity of 83.23% and specificity of 82.61% while the KTS had 88.02% and 73.91%, respectively, at a cutoff of 13 for predicting mortality. At admission, the GCS at a cutoff of 13 had sensitivity of 70.48% and specificity of 66.67% while the KTS had 68.07% and 62.50%, respectively, at a cutoff of 14 for predicting length of hospital stay.

**Conclusion:**

Comparatively, the GCS performed better than the KTS in predicting mortality and length of hospital stay. The GCS was also more accurate at labelling the head injury patients who died as severely injured as opposed to the KTS that categorized most of them as moderately injured. In general, the two scores were sensitive at detection of mortality and length of hospital stay among the study population.

## 1. Introduction

The term head injury is commonly used to describe injuries affecting not just the brain but also the scalp, skull, maxilla, mandible, and special senses of smell, vision, and hearing. Head injuries are also commonly referred to as brain injury or traumatic brain injury, depending on the extent of the head trauma [1]. In Uganda, head injury is one of the top four common admission diagnoses, contributing to a total 45.3% mortality rate in one study of intensive care unit patients and 75% head injury specific mortality rate in another study of all casualty admissions [2, 3]. Head injury was also found to be associated with 65% of all injury-related fatalities in urban Uganda [4]. With such high mortality rates, there is need for a clinical tool that is simple and easily reproducible that can be used to assess injury severity and more accurately predict mortality and length of hospital stay. In an international cohort study, trial results of 8937 patients predicted that a traumatic brain injury victim in low- and middle-income countries has twice the odds of dying after severe traumatic brain injury. Improvement in the assessment and prioritization of injuries has been shown to contribute to a 28% reduction in fatality rates in injury patients [5]. The Glasgow Coma Scale is a significant predictor of outcome following head injury. However, the prognostic value of the GCS is increased by taking other variables into account as well, such as mechanism of injury, age, head computed tomography (CT) findings, papillary abnormalities, and episodes of hypoxia and hypotension [6, 7]. The Kampala Trauma Score is a validated tool for assessing the severity of injury in low-resource settings [8–10]. Previous studies performed in low- and middle-income countries showed that both the GCS and the KTS were effective triage tools that could independently predict mortality and length of hospital stay. However, no study has been performed to compare the predictive value of the GCS and KTS for mortality and length of hospital stay among head injury patients at a tertiary hospital in low- and middle-income countries. Therefore, the present study aimed to assess injury severity in the study population using the two tools and to use these to compare their predictive value for mortality and length of hospital stay.

## 2. Materials and Methods

### 2.1. Study Setting

The study was carried out in Mulago National Referral Hospital at the Accident and Emergency (A&E) unit, Intensive Care Unit, Neurosurgery Ward, and Neurosurgery Outpatients Department. The hospital is situated in Kampala, 2 kilometres from the city centre, and serves as Makerere University College of Health Sciences Teaching Hospital and as a National Referral Hospital for Uganda. The hospital has an official bed capacity of 1790 beds. The A&E unit comprises of a surgical casualty area, a casualty theatre, a radiology unit that provides x-ray and ultrasound scan services, emergency laboratory, a pharmacy, a plaster room, emergency surgical ward for admitted patients, and a trauma centre to cater for severely injured patients. The neurosurgery ward currently has a 40-bed capacity divided into 5 high dependency unit beds and 35 general ward beds. It has four neurosurgeons and 33 nursing staff. The Intensive Care Unit at Mulago Hospital currently has a 7-bed capacity with attending intensivists, anaesthesiologists, and senior house officers from different departments and 22 nursing staff.

### 2.2. Study Design and Procedure

**Figure 1:**
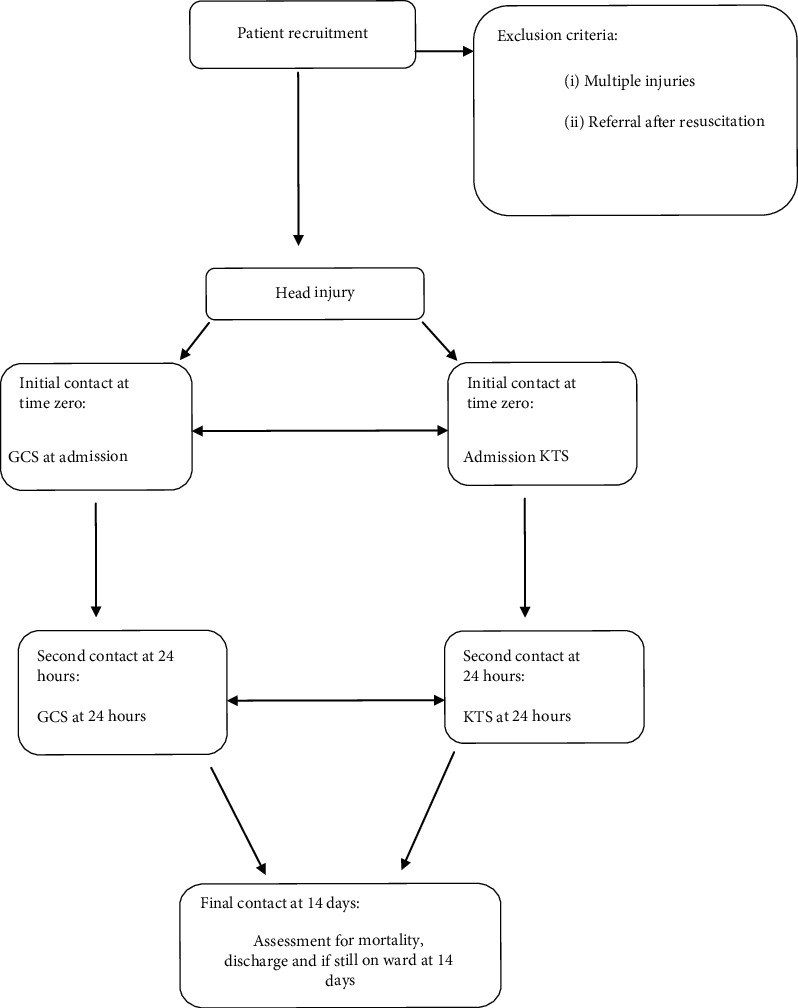
Flow chart.

This was a diagnostic prospective study. Head injury patients above 18 years admitted to the Accident and Emergency Unit in Mulago Hospital during the study period were solely eligible for recruitment. Patients who had head injury in addition to other injuries such as fractures of the femur, pelvis, haemoperitoneum, and haemothorax were excluded from the study. There was no restriction on patient inclusion in the study based on the time of injury and time of arrival to the hospital; however, those patients who had initial resuscitation in a different hospital before being referred to Mulago Hospital were not included in the study. Simple random sampling was performed to select the patients in the sampling frame with every second eligible participant considered therefore sampling at 50%. The data were collected every day of the week, 24 hours a day from January 12, 2018 to March 16, 2018. At admission, patients were screened for eligibility. Informed consent was sought at this point, and once this was obtained, a questionnaire was issued to the study participant. Admission time zero was marked on the questionnaire. This was defined as the time of initial interface between the clinician and the patient, and this was performed at the time of completion of the initial resuscitation. The admission time was used to establish the baseline GCS and KTS in the same patient that was to be compared to subsequent measurements; this was termed the admission GCS or KTS. The questionnaire was then filled. A second contact with the patient was at 24 hours following admission. Repeat measurements for the GCS and KTS were performed termed the GCS or KTS at 24 hours. The patients were followed up as they moved from the A&E unit either to the neurosurgery ward or the intensive care unit. The patients who were discharged before 14 days were followed through phone calls and reviewed in the neurosurgery out patients department to avoid loss to follow up and the outcome of interest determined. Final contact with the patient was at 14 days following admission. Whether the patient was alive or dead, their length of hospital stay, discharged home, transferred out, and if they are still in hospital 14 days post admission were assessed, and data were collected. The patients lost to follow up were subsequently excluded from the study.  Figure 1 illustrates a flow chart to show how patients were recruited into the study and then followed up until the end of the study.

### 2.3. Data Collection

The data collection was performed by four trained research assistants (nursing officers) who work at the emergency department of the hospital. The data were collected primarily, the GCS and KTS were determined by the research assistants, and the patients were also followed by them for 2 weeks.

### 2.4. Consent

Informed consent was sought for patients who were unconscious from their next of kin. Those patients who were unconscious without attendants were excluded from the study.

### 2.5. Statistical Analysis

All data were entered into EpiData version 4.2.0 and exported to STATA version 14.0 for analysis. Receiver operating characteristics (ROC) curves for the GCS and KTS as predictors of mortality at two weeks were constructed, and the area under the curve (AUC) based on nonparametric assumptions was generated for each GCS and KTS and compared. Similarly, ROC curves for prediction of hospitalization at two weeks were constructed. The GCS was compared to the KTS on the database using logistic regression. Odds ratio and 95% confidence interval were computed for each model. The two scores were again compared for sensitivity and specificity at particular cutoff points for prediction of mortality and length of hospital stay. The level of significance was set at *P* < 0.05.

### 2.6. Ethical Considerations

The research was approved by the Makerere University College of Health Sciences Research and Ethics Committee.

## 3. Results

### 3.1. Basic Demographic Information

The basic characteristics of patients with head injury included in the study are provided in Table 1.

Of these, 149 (78.4%) were male and 41 (21.6%) were female giving a male to female ratio of 3.6 : 1 (Table 1).

The median age was 30 years (interquartile range: 24–37), while the mean age was 32 years with a standard deviation (SD ± 11).

Of the three age groups, the 18–34 group contributed the greatest number of patients, that is, 129 (67.9%), with the 35–50 and >50 groups contributing 48 (25.3%) and 13 (6.8%), respectively (Table 1).

Majority of the patients had an education level up to the primary level, that is, 71 (37.4%), secondary 69 (36.3%), tertiary 16 (8.4%), and 34 (17.9%) had no formal education (Table 1).

### 3.2. Mechanism of Injury

**Figure 2:**
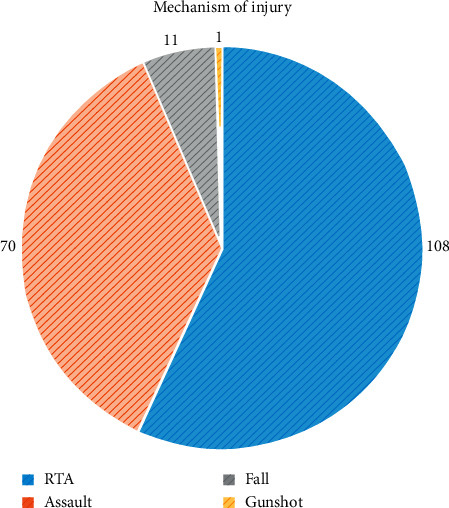
Mechanisms of injury.

The most common mechanism of injury was the road traffic accident (RTA), which contributed to 108 (56.8%) of all cases. Other mechanisms of injury included assaults (36.8%), falls (5.8%), and gunshot (0.5%) (Figure 2).

**Figure 3:**
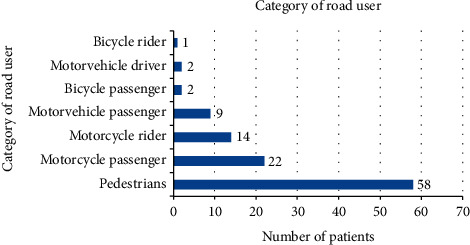
Categories of road users.

Of the 108 patients involved in road traffic accidents, 53.7% were pedestrians, 20.4% were motorcycle passengers, 13% were motorcycle riders, 8.3% were motor vehicle passengers, and motor vehicle drivers and bicycle passengers contributed 1.9% each, while bicycle riders accounted for 0.9% (Figure 3).

### 3.3. Categorization of Injury

Using the GCS and KTS, injured patients were classified as mild, moderate, or severe. The GCS and KTS were recorded at admission and at 24 hours.

The Glasgow Coma Scale classified the patients according to their severity of injury as follows. The mild injury with a score between 14 and 15, moderate injury with a score between 9 and 13, and severe injury with a score less than 9.

**Figure 4:**
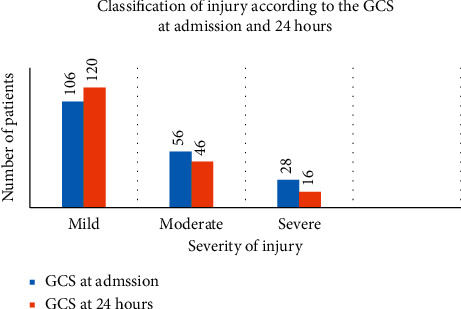
Classification of head injury according to the GCS at admission and at 24 hours.

The GCS at admission (*n* = 190) had 106 (55.8%) patients as mildly injured, while 56 (29.5%) and 28 (14.7%) were moderately and severely injured, respectively (Figure 4).

The GCS at 24 hours (*n* = 182) had 120 (65.9%) of patients as mildly injured, while 46 (25.3%) and 16 (8.8%) were moderately and severely injured, respectively (Figure 4).

The Kampala Trauma Score classified the patients according to their severity of injury as follows. The mild injury with a score between 14 and 16, moderate injury with a score between 11 and 13, and severe injury with a score less than 11.

**Figure 5:**
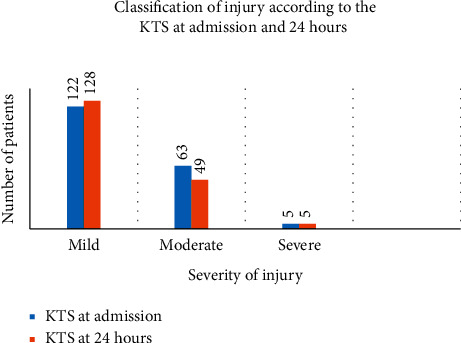
Classification of head injury according to the KTS.

The KTS at admission (*n* = 190) had 122 (64.2%) patients as mildly injured, while 63 (33.2%) and 5 (2.6%) were moderately and severely injured, respectively (Figure 5).

The KTS at 24 hours (*n* = 182) had 128 (70.3%) patients as mildly injured, while 49 (26.9%) and 5 (2.8%) were moderately and severely injured, respectively (Figure 5).

### 3.4. Outcomes

At the end of two weeks, of the 190 patients, 167 (87.9%) were alive and 23 (12.1%) were dead.

Of the 167 patients who were alive, 4 (2.4%) reached the ICU, 71 (42.5%) were admitted up to the neurosurgery ward, and 92 (55.1%) were admitted to the accident and emergency unit, and then, later discharged from there.

Of the 23 patients who died, 8 (34.8%) died from the neurosurgery ward and 15 (65.2%) died from the accident and emergency unit.

On the other hand, 166 (87.4%) patients spent less than 2 weeks in the hospital, and 24 (12.6%) patients spent more than two weeks in the hospital.

Of the 23 patients who died, according to the GCS classification, 15 (65.2%) had severe injury at admission, while according to the KTS classification, only 5 (21.7%) had severe injury at admission (Table 2).

### 3.5. Prediction of Mortality Using the GCS and KTS

**Figure 6:**
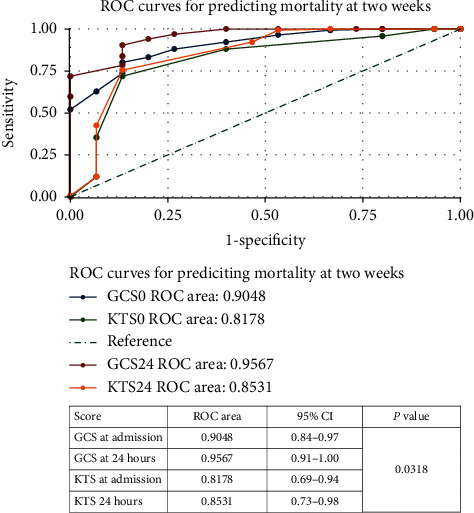
ROC curves comparing the GCS and KTS in predicting mortality at 2 weeks.

Figure 6 shows ROC curves for the GCS and KTS at admission and 24 hours as predictors of mortality at 2 weeks. The area under the curve (AUC) for GCS at admission is 0.9048 and at 24 hours is 0.9567. The area under the curve (AUC) for KTS at admission is 0.8178 and at 24 hours is 0.8531. The *P* value is 0.0318.

### 3.6. Predictions of Length of Hospital Stay Using the GCS and KTS

**Figure 7:**
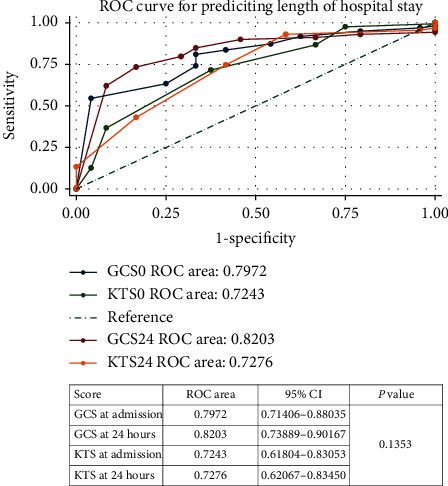
ROC curves comparing the GCS and KTS in predicting length of hospitalization.

Figure 7 shows ROC curves for the GCS and KTS as predictors of continuing hospitalization at 2 weeks. The accuracy of the test in predicting hospitalization at 2 weeks is poorer for all the scores (that is, ROC areas under the curve (AUC) further from 1.00). The GCS total areas under curve (AUC) of 0.7972 and 0.8203 at admission and 24 hours were compared to that of the KTS of 0.7243 and 0.7276, respectively. The *P* value is 0.1353.

### 3.7. Comparisons of Sensitivity and Specificity at Cutoff Points for the GCS and KTS in Prediction of Mortality and Length of Hospital Stay for Scores at Time Zero and 24 Hours

The sensitivity and specificity of the GCS and KTS as predictors of mortality and length of hospital stay at 2 weeks at different cutoff points were compared.

The sensitivity and specificity for the GCS and KTS at admission and at 24 hours were taken at cutoff points where the values of sensitivity and specificity were close to each other.

#### 3.7.1. Mortality

At a cutoff of 11, the GCS at admission had a sensitivity of 83.23% and a specificity of 82.61%. This is in comparison to sensitivity of 88.02% and 73.91% specificity for the KTS at admission at a cutoff of 13 (Table 3).

Conversely, at a cutoff of 12, the GCS at 24 hours had a sensitivity of 83.83% and specificity of 86.67%. This too is in comparison to a sensitivity of 75.45% and 86.67% specificity for the KTS at 24 hours at a cutoff of 14 (Table 3).

#### 3.7.2. Length of Hospital Stay

At a cutoff of 13, the GCS at admission had a sensitivity of 70.48% and specificity of 66.67%. This is in comparison to a sensitivity of 68.07% and 62.50% specificity for the KTS at admission at a cutoff of 14 (Table 4).

Conversely, at a cutoff of 13, the GCS at 24 hours had a sensitivity of 79.75% and 70.83% specificity. This is in comparison to sensitivity of 74.68% and 58.33% specificity for the KTS at 24 hours at a cutoff of 14 (Table 4).

The KTS alone was a strong predictor of mortality since it had a *P* value of 0.000. However, when the KTS is adjusted for the GCS, it does not remain an independent predictor of mortality with a *P* value of 0.133 (Table 5).

On the other hand, the KTS alone was still a strong predictor of length of hospitalization since it had a *P* value of 0.005. However, when the KTS is adjusted for GCS, it does not remain an independent predictor of length of hospitalization with a *P* value of 0.903 (Table 5).

The GCS alone and when adjusted for the KTS was a strong and independent predictor of mortality with a *P* value of 0.000 (Table 5).

Still the GCS alone and when adjusted for the KTS was a strong and independent predictor of length of hospital stay with a *P* value of 0.000 and 0.021, respectively (Table 5).

## 4. Discussion

### 4.1. Introduction

This study aimed to compare the predictive value of the GCS and KTS in head injury outcomes at Mulago National Referral Hospital. To determine which of the two tools is better at predicting mortality and length of hospital stay at 2 weeks.

### 4.2. Basic Demographics

The age and sex profile of this study is consistent with previous studies [6, 8, 11–13]. Males in their productive age contributing the greatest proportion of patients at 78.4% with the majority 129 (67.9%) in the 18–34 age group, which is consistent with the vastly young population in Uganda [14].

Road traffic accidents were the leading cause of injury, accounting for up to 56.8% of injuries as seen in most countries worldwide. This is similar to other studies where RTA was still the leading mechanism of injury from as low as 42% rising to as high as 79% in other studies [12, 13, 15, 16].

Only one case of gunshot injury was recorded (0.5%). A study in South Africa had three cases of gunshot wounds to the head [15].

### 4.3. Category of Injury

The GCS had 28 patients classified as severely injured at admission, while the KTS had 5 patients with severe injury. At 24 hours, the GCS classification had 16 patients as severely injured, while the KTS had 5 patients as severely injured. This can be attributed to the resuscitation and other care that the patients received that significantly reduced the number of patients classified as severely injured at admission and at 24 hours.

Most of the patients had mild injury; the GCS at admission had 120 with mild injury on the one hand and the KTS had 128 with mild injury on the other hand. This is in line with fact that the vast majority of patients who had RTA's were pedestrians who might have been knocked by vehicles or motorcycles that were not at top speed in the city traffic.

### 4.4. Outcomes

167 (87.9%) patients were alive and only 23 (12.1%) were dead at 2 weeks, which is also in line with the fact that most patients were classified to have mild injury and therefore were expected to survive even on follow up for 2 weeks.

The fact that the vast majority of patients had mild head injury also shows that Mulago Hospital is over burdened with patients who would be adequately treated at lower health facilities rather than being managed at the National Referral Hospital where we expect to receive only complicated or severe cases.

The overall mortality rate was 12.1%, which is consistent but slightly lower than has been previously reported in literature. This again could be can be explained by the fact that the vast majority of patients in this study only had a mild injury as opposed to other studies where they looked at patients that had severe head injuries only.

A study performed in Mbarara University Teaching Hospital had a mortality rate of 14% [17], while the mortality rate was found to be 25.8% in a study involving patients with severe head injury in Mulago Hospital [12].

According to the GCS classification of injury, the majority of patients who died (15/23 (65.2%)) had severe injury. According to the KTS classification, the patients who died (5/23 (21.7%)) had severe injury. The GCS is more accurate in defining patients as severely injured, and therefore, predicting the likelihood of mortality is based on the score as compared to the KTS that classified less patients as severely injured that still went on to die.

15 (65.2%) patients died from the casualty ward, while 8 (34.8%) died from the neurosurgery ward. The average survival time for the fatalities at the casualty ward was 15 hours, while the average survival time for the fatalities on the neurosurgery ward was days 6.7 days.

The vast majority of patients died from the casualty ward as opposed to the neurosurgery ward; these patients might have been severely injured and therefore had a poor prognosis. The Accident and Emergency Unit at Mulago Hospital is not solely a neurosurgical centre but also receives and manages patients with orthopaedic, general surgery, and other emergencies, thus leading to inadequate resources being allocated to the patients with head injury. This is in contrast to the neurosurgery ward where patients predominately have neurosurgery diagnoses.

Only 24 (12.6%) patients were hospitalized for more than 2 weeks, while the vast majority 166 (87.4%) were hospitalized for less than 2 weeks. This is also consistent with the fact that majority of the patients had a mild head injury; they are expected to stay in the hospital for a short time as opposed to those with severe injury who usually stay in the hospital for a longer time.

The ROC curve analysis demonstrated that for either prediction of mortality or continuing hospitalization at 2 weeks, the performance of the GCS was better than that of the KTS; however, the accuracy of the test in predicting hospitalization at 2 weeks was poorer for both the GCS and KTS, that is, ROC areas (AUC) further from 1.00.

### 4.5. Performance Assessment

The ROC shows the ability of the two scores to predict mortality and length of hospital stay in the study population based on area under curves (AUC).

#### 4.5.1. Prediction of Mortality at 2 Weeks

The GCS at admission and GCS at 24 hours (AUC 0.9048, 0.9567) provided the better prediction of hospital mortality than the KTS at admission and KTS at 24 hours (AUC 0.8178, 0.8531). The total areas under the curve for the KTS are similar to those in previous studies (0.836, 0.871) [9, 17], but still lower than previously noted in other literature (0.83–0.98) [18, 19].

#### 4.5.2. Prediction of Hospital Stay

The GCS at admission and GCS at 24 hours (AUC 0.7972, 0.8203) predicted length of the hospital stay better than the KTS at admission and KTS at 24 hours (AUC 0.7243, 0.7276). The ROC curves for prediction of hospitalization at 2 weeks (0.7243–0.7276) is better than the ROC curves in previous studies (0.647, 0.656) [9, 17].

#### 4.5.3. Comparison of GCS and KTS Sensitivity and Specificity

At admission, the GCS at a cutoff point of 11 was 83.23% sensitive in identifying those who died and 70.48% sensitive for identifying hospitalization at two weeks at a cutoff point of 13, in comparison to 88.02% and 68.07%, respectively, for the KTS at cutoff of 13 and 14.

At 24 hours, the GCS at a cutoff point of 12 was 83.83% sensitive in identifying those who died and 79.75% sensitive for identifying hospitalization at two weeks at a cutoff point of 13, in comparison to 75.45% and 74.68%, respectively, for the KTS at a cutoff of 14.

In general, the two scores were sensitive at detection of mortality and length of hospital stay among the study population.

## 5. Limitations

1. There might have been an interobserver variation in the scoring of the GCS and the KTS among the research assistants
2. The short length of follow-up of patients for only 2 weeks
3. Multiply injured patients who predominantly had head injuries but were not used as part of the sample size
4. No data were collected regarding patients that missed or declined to participate in the study; therefore, it is not possible to adequately determine how selection bias might have impacted the study.

## 6. Recommendations

The Glasgow Coma Scale in this study that looked at patients with solely head injury performed slightly better than the Kampala Trauma Score; however, further evaluation is still needed to assess which of these triage tools is better in patients with multiple injuries.

A study with a larger sample size and a longer duration of follow up for up to 6 months should be performed to identify longer term mortality and functional outcomes in these patients.

A study that compares the predictive value of the GCS and KTS for mortality and length of hospital stay in head injury patients with multiple injuries should be carried out as well.

## 7. Conclusions

The two scores quantitatively summarized injury severity and predicted the outcomes of mortality and length of hospital stay as less or more than 2 weeks in patients with head injury.

Both the GCS and KTS are easy to compute given the few parameters and the simple addition of scores. These tools can therefore be used to enhance quality medical service delivery to head injury patients through easier triage especially in low resource settings.

These tools can be useful in providing objective information to assess the prognosis of head injured patients based on the scores that they get such that patients with moderate to severe injury can be referred to tertiary centres for more specialized care.

Comparatively, the GCS performed better than the KTS in predicting mortality and length of hospital stay with greater total areas under the curve for each parameter. The GCS was also more accurate at labelling the head injury patients that died as severely injured as opposed to the KTS that categorized most of them as moderately injured.

## Figures and Tables

**Table 1:** Basic demographic information.

Characteristics	*n* = 190	%
Age (years)		
18–34	129	67.9
35–50	48	25.3
>50	13	6.8

Sex		
Female	41	21.6
Male	149	78.4

Education level		
Primary	71	37.4
Secondary	69	36.3
Tertiary	16	8.4
None	34	17.9

**Table 2:** Categorization of injury of dead patients (*n* = 23).

Category	GCS at admission	KTS at admission
Severe	15 (65.2%)	5 (21.7%)
Moderate	7 (30.4%)	16 (69.6%)
Mild	1 (4.4%)	2 (8.7%)

**Table 3:** Cutoff points for sensitivity and specificity for mortality.

Cutoff values	GCS	KTS
Admission	11	13
24 hours	12	14

**Table 4:** Cutoff points for sensitivity and specificity for length of hospital stay.

Cutoff values	GCS	KTS
Admission	13	14
24 hours	13	14

**Table 5:** Logistic regression.

Regression model	OR (95% CI) for odds of death	*P* value	OR (95% CI) for odds of hospitalization	*P* value
KTS	3.15 (2.08–4.78)	0.000	1.47 (1.12–1.94)	0.005
KTS adjusted for GCS	1.49 (0.89–2.52)	0.133	1.02 (0.69–1.53)	0.903
GCS	1.83 (1.49–2.25)	0.000	1.26 (1.11–1.42)	0.000
GCS adjusted for KTS	1.62 (1.26–2.09)	0.000	1.25 (1.03–1.50)	0.021

Findings are statistically significant if *P* value <0.05.

## Data Availability

The data used to support the findings of this study are available from the corresponding author upon request.

## Abbreviations

GCS: Glasgow Coma Scale
KTS: Kampala Trauma Score
CT: Computed tomography
A&E: Accidents and emergency.
